# Investigations on DNA damage and frequency of micronuclei in occupational exposure to electromagnetic fields (EMFs) emitted from video display terminals (VDTs)

**DOI:** 10.1590/S1415-47572010005000010

**Published:** 2010-03-01

**Authors:** NK Lakshmi, R Tiwari, SC Bhargava, YR Ahuja

**Affiliations:** 1 UGC Research Unit, Bhavan's New Science College, Hyderabad, Andhra PradeshIndia; 2 Department of Electrical and Electronic Engineering, Sree Nidhi Institute of Science and Technology, Hyderabad, Andhra PradeshIndia; 3 Genetics Unit, Vasavi Medical and Research Centre, Hyderabad, Andhra PradeshIndia

**Keywords:** electromagnetic fields (EMFs), video display terminals (VDTs), comet assay, DNA damage, micronuclei

## Abstract

The potential effect of electromagnetic fields (EMFs) emitted from video display terminals (VDTs) to elicit biological response is a major concern for the public. The software professionals are subjected to cumulative EMFs in their occupational environments. This study was undertaken to evaluate DNA damage and incidences of micronuclei in such professionals. To the best of our knowledge, the present study is the first attempt to carry out cytogenetic investigations on assessing bioeffects in personal computer users. The study subjects (n = 138) included software professionals using VDTs for more than 2 years with age, gender, socioeconomic status matched controls (n = 151). DNA damage and frequency of micronuclei were evaluated using alkaline comet assay and cytochalasin blocked micronucleus assay respectively. Overall DNA damage and incidence of micronuclei showed no significant differences between the exposed and control subjects. With exposure characteristics, such as total duration (years) and frequency of use (minutes/day) sub-groups were assessed for such parameters. Although cumulative frequency of use showed no significant changes in the DNA integrity of the classified sub-groups, the long-term users (> 10 years) showed higher induction of DNA damage and increased frequency of micronuclei and micro nucleated cells.

## Introduction

Electromagnetic technologies like personal computers and televisions have brought social and economic benefits to large sections of society. At the same time, their biological effects are raising concern due to the electromagnetic radiation emitted from video display terminals (VDTs). The video display units comprising cathode ray tube have a large number of applications in all spheres of life like communication and broadcasting, space research and medicine. There is a widespread apprehension that excessive exposure to these electromagnetic fields (EMFs) may hamper fundamental biological processes in the human body.

The potential effects of EMFs on human health vary widely depending on the frequency and intensity of the fields. In spite of years of research, there is still ongoing discussion whether radiofrequency (RF)-EMFs and extremely low frequency (ELF) -EMFs could induce any physiologically relevant effects (Krewski *et al.* 2007). The studies envisaging the possible health effects of EMF exposure at such field ranges have mainly focused on biological endpoints such as DNA damage (Lai and Singh 1996; Hook *et al.* 2004; Sun *et al.* 2006; Yao *et al.* 2008), increase in free radicals (Tice *et al.* 2002; Boland *et al.* 2002; Ferreira *et al.* 2006; Simko *et al.* 2006), induction of heat shock proteins (Lantow *et al.*, 2006; Sanchez *et al.*, 2007; Valbonesi *et al.* 2008) and cellular alterations (Kim *et al.* 2008; Schwarz *et al.* 2008).

Relatively less attention has been paid to health hazards from exposure to radiation in the intermediate EMFs, including the radiation emitted from personal computer cathode ray tube monitors, in the frequencies of 20 kHz. The workers are subjected to cumulative EMFs in their occupational environments comprising EMFs of 50 Hz powerline frequencies as well as 15-25 kHz RF-EMFs. Epidemiological studies have suggested that occupational exposure to VDTs is associated with increased risk of various health effects, particularly reproductive disorders, depression and cancer. However, the experimental and epidemiological data from the intermediate frequency (IF) range are sparse. Therefore, assessment of acute health risks in the IF range is currently based on known hazards at lower frequencies and higher frequencies.

The conflicting results have raised attention for further research on bioeffects of EMF fields taking into account exposure levels and duration. Apparently very few studies have documented genotoxicity in personal computer users. The present investigation reports DNA damage and chromosomal damage in peripheral blood lymphocytes of the exposed populations by alkaline comet assay or Single Cell Gel Electrophoresis (SCGE) and cytochalasin blocked micronuclei test (CBMN). To the best of our knowledge, there is no report from India on the genotoxic potential of occupational exposure to VDTs. Hence, this study was carried out to investigate the effect of occupational EMFs exposure on DNA damage and frequency of micronuclei in peripheral blood leukocytes of the VDT users. Analysis of the data was carried for all the exposed subjects pooled together as well as in sub-groups based on the duration and intensity effect of exposure.

## Subjects and Methods

###  Participants

The study included 138 subjects occupationally exposed to video display terminals for more than two years. The exposed subjects were screened along with 151 age, sex and diet matched controls with similar socioeconomic status. The exposed subjects were software professionals from software companies and consultancies in Hyderabad, India. In the detailed questionnaire, duration of exposure (years), frequency of exposure in hours/day were noted. Age, diet, gender, recent infection, smoking, drinking alcohol and exercise were also recorded for both exposed and unexposed populations.

###  Sampling

After taking informed consent, 2 mL peripheral blood was collected from each participant by venepuncture into heparinised disposable syringe and placed in ice to prevent exogenous damage. The sample was processed in the laboratory within an hour of collection for assessing DNA damage and micronucleus frequency.

###  Chemicals

The sources of chemicals were as follows: Agarose [low melting point (20 °C) and regular melting point (35 °C)], sodium lauryl sarcosinate, Triton X-100, silver nitrate (all from Sigma-USA); tungstosilicic acid(Koch-Light Laboratories, UK); sodium chloride, sodium hydroxide, potassium chloride, TRIS, EDTA, potassium dihydrogen phosphate and sodium phosphate dibasic (all from Glaxo, Mumbai, India); zinc sulphate and ammonium nitrate (Fisher, Madras, India); thiobarbituaric acid, butylated hydroxyl toluene, sulphosalicyclic acid and N-1-napthyl ethylene diamine dihydrochloride, potassium chloride, methanol, acetic acid (all from SD Fine Chemicals, Mumbai, India); RPMI-1640 media - Himedia, Phytohaemagglutinin – Gibco, Penicillin, Streptomycin - Himedia. Phytohaemagglutinin (PHA), Cytochalasin B (all from Sigma, USA), DMSO - (Merck, Germany).

###  Alkaline comet assay

Alkaline comet assay or single cell gel electrophoresis (SCGE) after Singh *et al.* (1998) was used to study DNA damage.

On a clean, dry, plain slide 100 μL of 0.75% normal melting agarose (NMA) prepared in phosphate buffered saline (PBS) was layered. These precoated slides were dried at 37 °C. On top of this layer, 30 μL of whole blood, mixed with 70 μL of 0.5% low melting agarose (LMA) prepared in PBS was layered. The third layer consisted of 100 μL of LMA. The slides were incubated in cold lysis buffer (2.5 M NaCl, 100 mM Na_2_ EDTA, 10 mM Tris; 1% sodium lauryl sarcosinate; 1% Triton X-100 and 10% DMSO added fresh) at 4 °C overnight.

The slides were removed from the lysing solution and placed side by side in a horizontal electrophoretic unit. The slides were completely immersed in freshly prepared alkaline electrophoretic buffer (1 mM Na_2_EDTA and 300 mM NaOH; pH 13) for 30 min to facilitate the DNA unwinding and expression of alkali labile sites. After alkali treatment, the electrophoresis was carried out for 30 min at 300 mA and 0.67 V/cm. The slides were carefully lifted from the buffer and gently washed with neutralizing buffer (0.4 M Tris buffer, pH 7.5). The slides were then washed with distilled water and air dried.

The air dried slides were immersed in the fixing solution (15% w/v trichloroacetic acid, 5% w/v zinc sulphate and 5% w/v glycerol) for 10 min and washed gently with double distilled water several times. For staining, 32 mL of staining solution A (5% w/v Na_2_CO_3_) was mixed with 68 mL of staining solution B (0.02% w/v NH_4_NO_3_, 0.02% w/v AgNO_3_, 0.1% w/v tungstosilicic acid and 0.05% v/v formaldehyde) and poured over the slides so as to cover the slides uniformly. This step was repeated until with a fresh mixture of staining solution a grayish colour developed on the slides. To stop staining, the slides were immersed in stopping solution (1% acetic acid) for 5 min, washed with double distilled water and air dried.

For visualization of DNA damage, a bright field, transmission light microscope (Leitz) was used at 400x magnification. Comet tail length was measured, using an ocular micrometer fitted in the eyepiece, in 200 cells per slide (in duplicate). Mean comet tail length, which is an estimate of DNA damage, was calculated for each sample.

###  Cytokinesis block micronucleus (CBMN) assay

Micronuclei (MN) were observed in cytokinesis-blocked cells using cytochalasin B (Cyt-B) following the method suggested by Fenech and Morley (1985).

About 0.2 mL of PHA was added to 5 mL of RPMI 1640 medium using 1 mL syringe. 15 drops of blood was added to each vial. Samples were initiated in duplicates. The culture vials were incubated for 72 h at 37 °C and shaken for proper mixing. Cyt-B (6 μg/mL) was added at 44^th^ hour after initiation of culture and incubated further for another 28 h at 37 °C and then cultures were harvested.

The cultures were centrifuged at 1000 rpm for 5-10 min. The supernatant was discarded and 5 mL of prewarmed hypotonic solution (0.56%) was added to the pellet drop by drop slowly on by vortexing and incubated for 10 min for 37 °C. Then the vials were centrifuged for a minute and supernatant was discarded. Cells were fixed in 5 mL of fixative (3:1 methanol: acetic acid) followed by two more changes of fixative.

The slides were prepared in triplicate by gently dropping the cell suspension onto the precleaned slides and flame dried. Slides were stained with 2% Giemsa for 10 min, rinsed and air dried.

To determine the MN yield, nearly 1000 binucleated (BN) cells were scored for each experimental condition under magnifications of 400x and finally 1000x from 2 coded slides/culture. Identification of MN was according to the criteria summarized by Countryman and Heddle (1976). Routinely on average ~2000 BN cells were scored for the presence of MN in each subject and mean values of the results were calculated.

###  Statistical analysis

The slides were coded during processing and decoded at the time of statistical analysis. For statistical evaluation, observations on each parameter for each group were pooled and mean ± SD was calculated. Student's t-test (paired and unpaired comparisons) and one way ANOVA were performed to evaluate various differences. Multiple regression analysis was done to study the effects of confounding factors and correlation analysis was carried out to adjudge the sensitivity of parameters used. Statistical software SPSS 15 was used to carry out statistical analysis.

## Results

The general characteristics of the study group and controls are shown in Table 1. The mean (±SD) duration of VDT use was 7 (±4.8) years with a mean (±SD) cumulative frequency of 389 (±147) minutes per day.

The results of basal DNA damage assessed by alkaline comet assay in terms of mean comet tail length ±SD are summarized in Table 2. Independent *t* test showed no significant difference in the mean comet tail length values of exposed and controls. The results of CBMN assay on binucleated cells, percentage micronuclei (%MN) and percentage micronucleated cells (%MNC) of the exposed groups are shown in Table 3. Overall, there was no significant difference in the frequency of micronuclei between the exposed subjects and the controls (Table 3). However, results from one-way ANOVA revealed significant differences in DNA damage and incidence of micronuclei among sub-groups based on duration of exposure (years) of exposed subjects (Table 4). The significant mean comet tail length and incidences of micronuclei was observed in sub-group having duration of exposure more than 10 years. The two sub-groups based on frequency of exposure (< 420 min/day and > 420 min/day) had no significant difference in damage levels (Table 5).

Pearson's coefficient of correlation was carried out between different parameters to assess the extent of relationship between the endpoints of comet assay and CBMN assay (Table 6). The mean comet tail length and frequency of micronuclei showed no significant correlation in the exposed as well the control subjects. Of the various confounding factors studied, no significant effect could be seen in multiple regression analysis with respect to gender, age and habits.

## Discussion

Non-panel video display screens of computer monitors produce significant EMFs despite improvements in technology over the last decade or so. In India presently there is a boom in Information Technology (IT) and Business Process Outsourcing centers. The IT sector witnessed considerable activity since 2004 including a ramping up of operations by major multinational corporations. Apart from the powerline frequencies (ELF-EMFs) the software employees are exposed to EMFs emitted from personal computers.

The workers are subjected to cumulative EMFs in their occupational environments. To the best of our knowledge, the present study is the first attempt to carry out multiple markers on assessing bioeffects in subjects occupationally exposed to Cathode Ray Tube (CRT) from personal computers. The aim of our study was to investigate genotoxicity in workers occupationally exposed to CRT VDTs through the induced DNA damage and micronuclei in leukocytes and lymphocytes respectively.

Overall, the results on DNA damage and micronuclei frequency showed no significant differences. With exposure characteristics, such as total duration (years) and frequency of use (minutes/day), sub-groups were also assessed for such parameters. The long-term users (> 10 years) showed higher induction of DNA damage and increased frequency of micronuclei and micronucleated cells. The cumulative frequency of use showed no significant changes in the DNA integrity between the classified sub-groups.

The subjective symptoms reported by the PC usage include predominantly headache, followed by sleeplessness and neck pain. Duration and intensity was not significant predicting factors for the reported symptoms. Very few *in vivo* studies have directly evaluated the cytogenetic damage in computer workers. Carbonari *et al.* (2005) indicated significant cytogenetic damage by micronuclei assay in computer workers, reinforcing the data obtained in our study. Estecio and Silva (2002) also evaluated possible nuclear alterations in microcomputer's workers and found that exposed individuals had two times more chromosomal aberrations in cultured lymphocytes than control individuals. The rationale behind such findings have been proposed to be the genotoxic influences of EMFs may be through increased free radical activity or acceleration of electron transfer in different enzymes and proteins.

In the present study, no thermal effects of EMFs could be seen. However, Gangi and Johansson (2000) detected an increased incidence of skin and central nervous system (CNS) alterations among microcomputer workers. Andersson (1996) reported that female workers exposed to VDU presented obstetric complications, besides skin, ocular and CNS diseases.

Thus we conclude that this epidemiological study on occupational EMF exposure was significant to provide preliminary evidence on health risk along with the pertinent reliable parameters carried out as biomarkers. The long-termed effect also implicated the need for close monitoring of health hazards associated in long-term VDT users.

## Figures and Tables

**Table 1:** General characteristics of the study group and controls.

S. n.	Variables	Exposed subjects	Control
1	Number(*n*)	138	151
2	Age (range)	21-47	17-44
	(Mean ± SD)	25.13 ± 4.07	22.88 ± 4.45
3	Sex		
	Males	87 (63.04%)	101 (66.89%)
	Females	51 (36.96%)	50 (33.11%)
4	Smoking habits		
	Smokers	32 (23.2%)	0 (0%)
5	Drinking habits		
	Alcoholism	34 (24.6%)	0 (0%)
6	Dietary habits		
	Vegetarian	53 (38.41%)	74 (49.01%)
	Non-vegetarian	85 (61.59%)	77 (50.99%)

**Table 2:** Mean ± SD values of DNA damage in exposed and control subjects.

Parameters	Exposed subjects			Control		t value	p value
	N	Mean ± SD		N	Mean ± SD		
Comet tail length (arbitrary units)	138	3.76 ± 1.38		151	3.69 ± 1.13	1.895	0.061^NS^

^NS^: Non-significant at 5%.

**Table 3:** Mean ± SE values of frequency of micronuclei in exposed and control subjects.

Groups	Sample size	Number of BN cells	Total number of micronuclei	Mean ± SE of MN cells (%)	Mean ± SE of MN (%)
IF-EMF	34	35053	413	1.16 ± 0.48^NS^	1.39 ± 0.63^NS^
Control	60	64026	661	1.04 ± 0.52^NS^	1.24 ± 0.67^NS^

^NS^: Non-significant at 5%.

**Table 4:** DNA damage and frequency of micronuclei in relation to duration of exposure to VDTs.

Observation		Group based on duration of exposure (years)		t value	p value
		1-6	> 7		
CTL	N	73	65	21.96	< 0.001
	M	3.15 ± 0.24	4.09 ± 0.26		

%MNC	N	20	14	28.39	< 0.001
	M	1.00 ± 0.19	2.73 ± 0.15		

%MN	N	20	14	5.80	< 0.001
	M	1.19 ± 0.24	1.63 ± 0.18		

CTL-comet tail length, MN- micronuclei, MNC- micronucleated cells, N- sample size, M-mean ± SE.

**Table 5:** DNA damage and incidences of micronuclei in relation to frequency of exposure (minutes/day) to VDTs.

Observation		Group based on frequency of exposure (minutes/day)		t value	p value
		A 180-420	B 420-720		
CTL	N	64	74	1.259	0.210
	Mean ± SE	3.5991 ± 0.16	3.8914 ± 0.17		

%MNC	N	20	14	1.177	0.249
	Mean ± SE	1.2382 ± 0.11	1.0474 ± 0.12		

%MN	N	20	14	1.026	0.317
	Mean±SE	1.5085 ± 0.14	1.2526 ± 0.16		

CTL-comet tail length, MN- micronuclei, MNC- micronucleated cells, N- sample size, M-mean ± SE.

**Table 6:** Pearson's correlation analysis in exposed and control subjects.

Group	CTL	% MN	% MNC
Exposed			
CTL		0.002	0.026
%MN	0.002		0.977**
%MNC	0.026	0.977**	

Control			
CTL		0.03	0.04
%MN	0.03		0.70**
%MNC	0.04	0.70**	

**Correlation is significant at the 0.01 level (2-tailed).
